# Bundling the removal of emerging contaminants with the production of ligninolytic enzymes from residual streams

**DOI:** 10.1007/s00253-022-11776-7

**Published:** 2022-01-25

**Authors:** Sandra González-Rodríguez, Thelmo A. Lu-Chau, Alba Trueba-Santiso, Gemma Eibes, María Teresa Moreira

**Affiliations:** CRETUS, Department of Chemical Engineering, Universidade de Santiago de Compostela, Galicia 15782 Santiago de Compostela, Spain

**Keywords:** Manganese peroxidase, *Irpex lacteus*, Wheat straw, Emerging contaminants, Enzymatic oxidation

## Abstract

**Abstract:**

Enzymes offer interesting features as biological catalysts for industry: high specificity, activity under mild conditions, accessibility, and environmental friendliness. Being able to produce enzymes in large quantities and having them available in a stable and reusable form reduces the production costs of any enzyme-based process. Agricultural residues have recently demonstrated their potential as substrates to produce ligninolytic enzymes by different white rot fungi. In this study, the biotechnological production of a manganese peroxidase (MnP) by *Irpex lacteus* was conducted through solid-state fermentation (SSF) with wheat straw as substrate and submerged fermentation (SmF) employing wheat straw extract (WSE). The obtained enzyme cocktail also showed manganese-independent activity (MiP), related to the presence of a short MnP and a dye-decolorizing peroxidase (DyP) which was confirmed by shotgun proteomic analyses. In view of the enhanced production of ligninolytic enzymes in SmF, different parameters such as WSE concentration and nitrogen source were evaluated. The highest enzyme titers were obtained with a medium formulated with glucose and peptone (339 U/L MnP and 15 U/L MiP). The scale-up to a 30 L reactor achieved similar activities, demonstrating the feasibility of enzyme production from the residual substrate at different production scales. Degradation of five emerging pollutants was performed to demonstrate the high oxidative capacity of the enzyme. Complete removal of hormones and bisphenol A was achieved in less than 1 h, whereas almost 30% degradation of carbamazepine was achieved in 24 h, which is a significant improvement compared to previous enzymatic treatments of this compound.

**Key points:**

• *Wheat straw extract is suitable for the growth of I. lacteus.*

• *The enzyme cocktail obtained allows the degradation of emerging contaminants.*

• *Mn-dependent and Mn-independent activities increases the catalytic potential.*

**Graphical abstract:**

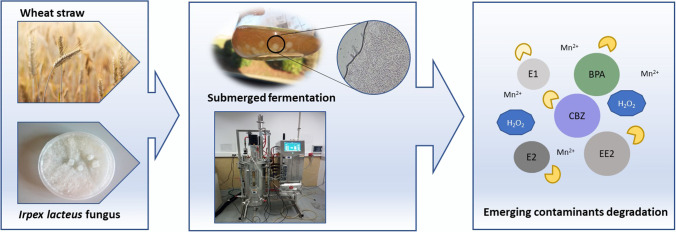

**Supplementary Information:**

The online version contains supplementary material available at 10.1007/s00253-022-11776-7.

## Introduction

White rot fungi (WRF) include a broad spectrum of fungal species that produce ligninolytic enzymes such as laccases, lignin peroxidase (LiP), manganese peroxidase (MnP), and other peroxidases, whose main function is lignin mineralization (Yadav and Yadav 2015). These fungi are widely known for their great applications in different fields, such as food industry, agriculture, chemicals, bioremediation of emerging pollutants, and pharmaceutical industry (Solomon et al. 2019). The versatility of these fungi lies in the great diversity of the enzymes, which are able to attack a wide spectrum of compounds including phenolic, methyl, and sulfonic groups (Bankole et al. 2019; Venkatesagowda 2019). These enzymes can generate highly reactive and non-specific radicals involved in the degradation of recalcitrant compounds, such as dyes and xenobiotics (Kersten and Cullen 2007; Godoy et al. 2016; Sosa-Martínez et al. 2020). Among WRF, *Irpex lacteus* has shown a significant role in different biotechnological fields due to the production of extracellular oxidative and hydrolytic enzymes (Novotný et al. 2009).

There are two options for the development of bioremediation processes, either in vivo, in which the fungus is cultivated in the presence of the target pollutants, or in vitro, in which it is necessary to consider a previous stage of enzyme production. Considering the implications of this procedure, the second option is much simpler to avoid contamination by competing microorganisms that inhibit the growth of the main culture. One of the constraints of biotechnological production is the culture medium cost, so it is particularly interesting to use waste streams as nutrient sources to reduce production expenses. Considering the fact that *I. lacteus* can degrade residual lignocellulosic biomass (e.g., corn stover, barley straw, corncob, and wheat straw) (García-Torreiro et al. 2016), such streams may also be good candidates as substrates for fungal growth. Although there are many studies using wheat straw as a solid substrate for the production of ligninolytic enzymes by WRF (Ćilerdžić et al. 2017; Gupta and Jana 2018, 2019), to our knowledge, there are no studies of peroxidase production by *I. lacteus* using a wheat straw extract as a substrate.

Genome analysis reveals that the main ligninolytic enzyme of *I. lacteus* is MnP, with a major role in lignin degradation (Yao et al. 2017). However, depending on the substrates used and fermentation conditions, other ligninolytic enzymes, such as versatile peroxidase (VP) and laccase, are also produced (Rothschild et al. 2002). For example, Salvachúa et al. (2013b) described the joint production of peroxidases with Mn(II)-dependent and Mn(II)-independent activities in fermentations with *I. lacteus*, which increases the range of use of its enzyme cocktail in different biodegradation processes.

The oxidative potential of MnP has been key in its ability to oxidize polycyclic aromatic hydro-carbons, chlorophenols, industrial effluents (mainly from the pulp and paper industry), and the bioremediation of contaminated soils (Saroj et al. 2013). The catalytic cycle of this enzyme is similar to that of other peroxidases, since it involves a two-electron oxidation. Furthermore, MnP is capable of oxidizing Mn^2+^ to Mn^3+^, which is a diffusible oxidant capable of penetrating the cell wall matrix and degrading phenolic substrates (Hofrichter 2002). Otherwise, the Mn(II)-independent activity of some peroxidases also allows the oxidation of different compounds with a similar structure to synthetic dyes (Fraaije 2012), but with the advantage of not requiring the presence of manganese, which simplifies the biodegradation process. However, the major drawbacks of these enzymes for a wide industrial application are their high production cost and their limited yield (Elisashvili and Kachlishvili 2009). Therefore, it is important to find new ways to obtain these enzymes at a lower cost, which highlights the convenience of crude rather than purified enzymes (Cardoso et al. 2018; Wang et al. 2020).

From the point of view of the fungal culture, two options can be considered: submerged fermentation (SmF) or solid-state fermentation (SSF). The former has been applied to a wider range of processes since the design, operation, and control of the fermenter are relatively simple (Musoni et al. 2015). These systems are homogeneous from the point of view of cell concentration, nutrients, and products. However, SSFs also offer some advantages, such as less effort in downstream processing, low costs and energy, lower sterility demands, and similar culture conditions to those of the natural fungal habitat, but, with the negative aspects of heterogeneity, limited mixing and oxygen transfer (Hölker et al. 2004; Couto and Sanromán 2006). A preliminary evaluation of both alternatives for the use of solid waste streams such as wheat straw finds in SSF an interesting solution to valorize this type of streams and minimize the generation of waste streams and water consumption.

Emerging contaminants (ECs), including pharmaceutical compounds such as hormones and endocrine-disrupting chemicals such as bisphenol A (BPA), are in the spotlight due to their negative impact on the environment. Their worldwide widespread use results in the presence of these compounds and their derivatives in wastewater at concentrations between ng to µg/L (Tran et al. 2018). For this reason, this study evaluates the degradation kinetics of different ECs using the enzymatic crude obtained in SmF and analyzing their biotransformation under different reaction conditions, including the enzyme dose and the rate of H_2_O_2_ addition. The present work does not focus on the purification of the enzymes produced, but on the use of an enzyme cocktail with potential application in the degradation of ECs. The use of a waste stream as a substrate for fermentation can lead to satisfactory production yields and lower costs.

## Materials and methods

### Chemicals and raw material

Carbamazepine (CBZ), BPA, estrone (E1), 17b-estradiol (E2), 17a-ethinylestradiol (EE2), MnSO_4_, C_2_H_3_NaO_2_, H_2_O_2_, 2,6-dimethoxyphenol (DMP), and Tween-80 were purchased from Sigma-Aldrich (Barcelona, Spain). Glucose, KH_2_PO_4_, NaC_2_H_3_O_2_, and CaCl_2_ were purchased from Panreac (Barcelona, Spain), meat peptone from Cultimed (Barcelona, Spain), yeast extract from iNtRON Biotechnology (Seongnam, South Korea), and MgSO_4_·7H_2_O from Fluka (Steinheim, Germany). Wheat straw was obtained from a local supplier (Carral, A Coruña, Spain) and stored at room temperature until use. The contents of cellulose and hemicellulose, as well as fermentable sugars in the wheat straw, are presented in Supplemental Table S1. These parameters are decisive in identifying the concentration of the C source present in the wheat straw for the formulation of the fermentation medium.

### Fungal strain and inoculum preparation

The fungus *I. lacteus*, strain Fr. 238 617/93 was obtained from the Culture Collection of Basidiomycetes (CCBAS) of the Academy of Sciences of the Czech Republic, Prague. For mycelium production in static cultures, three plugs of active mycelia were transferred from Petri dishes to Fernbach flasks with 100 mL of Kimura medium (glucose 20 g/L, peptone 5 g/L, yeast extract 2 g/L, KH_2_PO_4_ 1 g/L, MgSO_4_·7H_2_O 0.5 mg/L) and maintained at 30 °C for 7 days. After this step, the contents of the Fernbach flasks were crushed in a blender and used as inoculum for the Erlenmeyer flasks.

### Fermentations

#### SSF

Wheat straw was cut into 2 cm particles before being used as substrate and support for fungal growth. To evaluate whether wheat straw was sufficient for fungal growth, SSF was performed with two different media, one with wheat straw moistened with distilled water and the other with wheat straw moistened with a solution of glucose and peptone (4 g/L glucose and 1 g/L peptone). Each 250 mL Erlenmeyer flask containing 3 g of wheat straw and 9 mL of culture medium was sterilized for 45 min at 110 °C (RAYPA AES-75, Barcelona, Spain) and inoculated with 1 mL of inoculum. The Erlenmeyer flasks were incubated for 21 days at 30 °C in a humidity-saturated environment under static conditions. The initial pH of the medium in all experiments was 4.5. All experiments were conducted in duplicate.

For enzyme harvesting, the contents of each Erlenmeyer flask were extracted with 30 mL of distilled water. The Erlenmeyer flasks were then shaken in an orbital shaker (C24 Incubator Shaker, New Brunswick Scientific, Edison, New Jersey, USA) for 1 h at 175 rpm to facilitate the separation of the enzyme adsorbed on the wheat straw. The harvested liquid was then filtered through a cellulose filter and centrifuged for 10 min at 10,000 rpm prior to the determination of enzyme activity in the supernatant.

#### SmF with wheat straw extract (WSE)

WSE was obtained by adding 75 g of wheat straw to 1 L of distilled water, autoclaving the mixture at 121 °C for 20 min, and finally removing the solids by filtration. The WSE composition is shown in Supplemental Table S2. To assess whether the concentration of WSE had any impact on enzyme production, experiments were carried out with the original extract and with a diluted solution of the extract (25% in distilled water). Both media were supplemented with glucose and peptone (or casein hydrolysate) as shown in Table 1. The addition of Tween-80 (0.5 mL/L) as a surfactant was intended to enhance enzyme production and avoid problems associated with substrate hydrophobicity.

**Table 1:** Supplementation of the different media elaborated with diluted and original WSE

	Glucose (g/L)	Peptone (g/L)	Casein hydrolysate (g/L)	Tween-80 (mL/L)
Original WSE (100%)	4	1	-	0.5
	4	-	1	0.5
Diluted WSE (25%)	4	1	-	0.5
	4	-	1	0.5

Erlenmeyer flasks containing 90 mL of the final medium were autoclaved at 110 °C for 45 min and inoculated with 10 mL of inoculum. The Erlenmeyer flasks were incubated on an orbital shaker (Innova 4000 Incubation Shaker, New Brunswick Scientific, New Jersey, USA) at 30 °C and 150 rpm for 7 days. Fermentations were carried out in triplicate with an initial pH of 4.5. The initial pH was selected based on previous works with WRF (Mäkelä et al. 2013; Lú-Chau et al. 2018) and aiming to promote the activity and stability of MnP (Qin et al. 2014). The sampling strategy considered the periodic withdrawal of 3 mL of culture medium under sterile conditions, which were centrifuged for 10 min at 10,000 revolution per minute (rpm) to remove fungal biomass. The supernatant was used to monitor the culture pH, to determine glucose concentration, and to analyze MnP and MiP activities. The initial glucose concentration in all SmF fermentations was higher than the supplemented 4 g/L, due to the remaining glucose from the inoculum.

#### SmFs on conventional media

To compare the enzyme production using wheat straw with another conventional media, two of the most typical culture media were tested: Kimura medium (see “Fungal strain and inoculum preparation” section) and modified Kirk medium (glucose 10 g/L, ammonium tartrate 0.5–10 g/L, sodium acetate 2.72 g/L, KH_2_PO_4_ 2 g/L, CaCl_2_ 100 mg/L, mineral salts 10 mL/L, thiamine 2 mg/L, MnSO_4_ 84.51 mg/L) at pH 5.5.

#### Reactor operation

The scale-up of enzyme production by *I. lacteus* under SmF was carried out in a 30 L stirred tank bioreactor (Biostat Cplus, Sartorius Stedim Biotech SA, Goettingen, Germany). The fungus grown in Erlenmeyer flasks was used as inoculum at 10% v:v. The medium was prepared with diluted WSE (25% in distilled water) and supplemented with 4 g/L of glucose and 1 g/L of peptone at pH 4.5.

The reactor was operated for 3 days with temperature (30 °C), agitation (150 rpm), and air flow (10 L/min) control. Foaming was automatically controlled by adding antifoam (Antifoam 204, A6426, Sigma-Aldrich, St Louis, MO, USA) through a peristaltic pump. Other parameters measured on-line were redox potential, dissolved oxygen, and pH.

The culture broth collected at maximal MnP and MiP activities was filtered through a filter paper (Whatman No.1, Maidstone, UK). Afterwards, it was passed through a microfiltration membrane (Filtron Minisette System, Pall Corporation, Hauppauge, NY, USA; 0.2 µm cut-off) and finally concentrated by ultrafiltration with a 10 kDa membrane (Filtron Minisette System, Pall Corporation, Hauppauge, NY, USA). The cell-free concentrated crude, designated as enzymatic cocktail, was stored at −20 °C.

### Protein gel electrophoresis

The presence of different enzymes in the enzyme cocktail was confirmed separating them by sodium dodecyl sulfate-polyacrylamide gel electrophoresis in denaturing conditions (SDS-PAGE). For this, the protein concentration of the enzyme cocktail was estimated employing a Pierce™ BCA Protein Assay Kit (ThermoFisher Scientific, Waltham, MA). Electrophoresis was run for triplicate aliquots with 17 ng protein/well. As molecular weight marker, the PageRuler™ Plus Prestained Protein Ladder from 10 to 250 kDa (ThermoFisher Scientific) was included. Bis-Tris NuPAGE 4–12% gel was used (ThermoFisher Scientific) and electrophoresis was run at 200 V.

### Shotgun proteomic analysis by mass spectrometry

Concentrated enzyme cocktail was processed in solution by trypsin digestion, reduction-alkylation, and finally desalted using ZipTip-μC18 material (Merck Millipore, Burlington, MA). The obtained sample was analyzed using the nanoUHPLC-Tims-QTOF technique. Peptide samples (0.3 µg of protein) were injected onto a timsTOF Pro (Bruker, Bremen, Germany) equipped with a nano-electrospray source (CaptiveSpray) and a tims-QTOF analyzer. The chromatographic analysis was performed using a nanoELUTE chromatograph (Bruker) with a ReproSil C18 column (50 × 0.075 mm, 1.9 µm, 120 Å, Bruker). The nHPLC was configured with binary mobile phases that included solvent A (0.1% formic acid in ddH_2_O) and solvent B (0.1% formic acid in acetonitrile). The analysis time was 20 min, in which B/A solvent ratio was gradually increased.

For MS acquisition, a CID fragmentation and a nanoESI positive ionization mode was employed. PASEF-MSMS scan mode was stablished for an acquisition range of 100–1700 m/z. MS/MS spectra were processed with PEAKS Studio (Bioinformatics Solutions, Waterloo, ON) software for protein identifications using a homemade database with all protein sequences available in NCBI protein database for *I. lacteus* taxonomy. The label-free module from PEAKS Studio was used for protein semiquantification.

### Enzymatic removal of ECs

The enzymatic removal of CBZ, BPA, E1, E2, and EE2 was carried out in batch experiments using the enzyme cocktail produced by *I. lacteus* in the 30 L fermenter. These experiments were performed in 100 mL Erlenmeyer flasks sealed with Teflon stoppers, with continuous agitation at 150 rpm (orbital shaker SSM1, Stuart, Stone, UK), and at room temperature (22 ± 2 °C). The reaction mixture (50 mL) was composed of a mixture of the five ECs (300 µg/L each), MnP (50–200 U/L), MnSO_4_ (1 mM), and sodium malonate (50 mM) at pH 4.5. This pH was selected based on previous works evaluating the degradation of emerging pollutants by peroxidases (Eibes et al. 2011; Moon and Song 2012; Méndez-Hernández et al. 2015; Taboada-Puig et al. 2015, 2016).

The reaction was initiated by the continuous addition of H_2_O_2_ (4.3 and 42.9 H_2_O_2_ mM stock) at a rate of 1–10 µM/min (referred to the reaction volume) with a syringe pump (Multi-PhaserTM NE-4000 double syringe pump, New Era Pump Systems Inc, Farmingdale, USA) operating at the constant flow rate of 11.65 µL/min. In addition, controls for each experiment were performed with thermally inactivated enzyme to confirm that H_2_O_2_ cannot degrade ECs in experiments lacking enzyme. Samples were taken at regular intervals to measure MnP and MiP activities. The reaction was stopped by adding methanol (50% v/v) and stored at −20 °C for subsequent analysis of the ECs concentration. Each condition was carried out in triplicate.

### Analytical protocols

Glucose concentration was determined by using an enzymatic quantitative kit (GLUCOSE-TR, Spinreact, Girona, Spain). MnP activity was measured by monitoring the oxidation of DMP in the presence of Mn^2+^ (1 mM) at 30 °C and pH 4.5. MiP activity was determined as above but lacking Mn^2+^. One unit of enzyme activity was defined as the amount of enzyme forming 1 μmol of product per minute. Changes in absorbance over time were monitored at 468 nm using a UV-visible spectrophotometer (Shimadzu UV-1800, Shimadzu, Europa GmbH, Duisburg, Germany). Laccase activity was measured by 2,2′-azino-bis(3-ethylbenzothiazoline-6-sulfonic acid) (ABTS) oxidation following the protocol described by Muñiz-Mouro et al. (2017).

The concentrations of the target contaminants were determined by HPLC (Jasco XLC HPLC, Jasco Analitica España, Madrid, Spain) equipped with a 3110 MD diode array detector and a reverse phase column (150 × 4.6 mm, particle size: 3 μm) (Gemini, Phenomenex, Jasco Analitica España, Madrid, Spain). The operational conditions for ECs analysis were 100 µL injection volume, λ 210 nm, the eluent acetonitrile:H_2_O (55:45), and 35 °C. Retention times were 3.3, 4, 4.5, 5, and 5.4 min for CBZ, BPA, E1, E2, and EE2, respectively. The quantification limits for all ECs were 10 µg/L.

## Results

### Solid-state fermentations

**Fig. 1:**
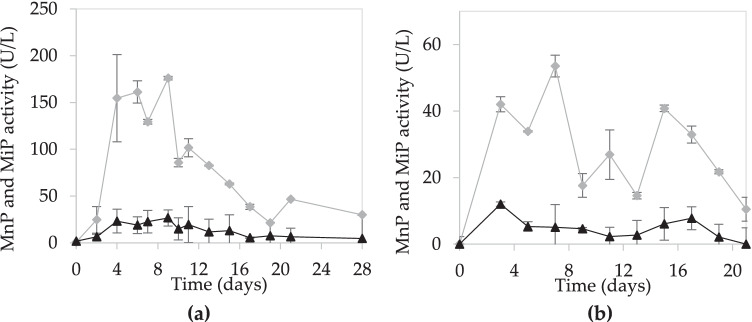
Enzyme activities during SSFs: **a** MnP (  ) and MiP (▲) activities in the culture with wheat straw as only C source; **b** MnP (  ) and MiP (▲) activities in the culture with wheat straw, glucose, and peptone

Wheat straw was used as substrate and support for the SSF of *I. lacteus*. The additional supply of glucose (4 g/L) and peptone (1 g/L) as carbon and nitrogen sources was evaluated in terms of manganese peroxidase production (Fig. 1). MnP activity was higher in the SSF without supplementation (NS-SSF, 176.0 ± 8.6 U/L), and the titers achieved were significantly higher than those of the SSF supplemented with glucose and peptone (GP-SSF, 53.6 ± 11.8 U/L). It is also noteworthy that the time required to reach the maximum production was similar in both SSF, being 9 days in the NS-SSF and 7 days in the GP-SSF. Once the maximum activity was reached, a decrease in MnP activity was observed. On the other hand, the maximum MiP activities (26.6 ± 2.8 and 12.1 ± 2.3 U/L) were reached on day 3 and day 9 for the SSF without and with extra nutrient addition, respectively. However, the MiP activity profile of NS-SSF shows production peaks similar to the maximum in earlier stages of the fermentation.

### Production of MnP in submerged fermentations

#### SmF on conventional media

In order to compare enzyme production with WSE-based media, fermentations of *I. lacteus* were carried out using two of the most common conventional media: Kirk and Kimura (Tien and Kirk 1988; Kimura et al. 1990). In these experiments, only the Mn(II)-dependent activity was studied. Table 2 shows the MnP production throughout the 14-day fermentation. The highest MnP activity (100.3 ± 26.5 U/L) was reached in Kimura medium on day 8, while in the case of Kirk medium, the maximum production peak (60.4 ± 6.7 U/L) was reached on day 14.

**Table 2:** MnP activity (U/L) during SmF on conventional media

	Day 2	Day 4	Day 6	Day 8	Day 10	Day 12	Day 14
Kirk medium	18.5 ± 0.5	22.6 ± 2.0	46.0 ± 11.8	45.4 ± 14.4	51.7 ± 15.2	51.7 ± 2.5	60.4 ± 6.7
Kimura medium	17.3 ± 2.2	38.2 ± 2.2	57.2 ± 8.0	100.3 ± 26.5	98.4 ± 18.4	31.0 ± 8.7	22.3 ± 1.9

#### SmF using wheat straw extract supplemented with glucose and peptone

**Fig. 2:**
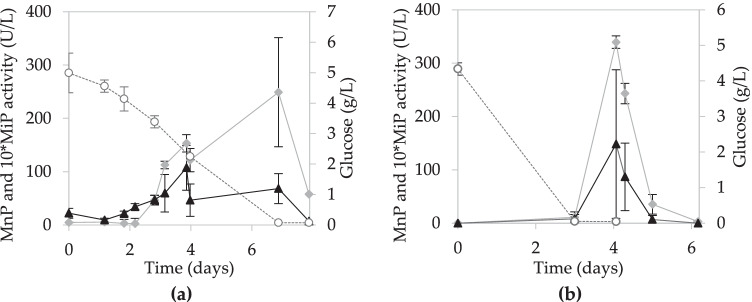
Evolution of MnP activity (  ), MiP activity (▲), and glucose concentration (○) in the SmF with **a** original and **b** diluted WSE supplemented with glucose and peptone

Submerged fermentation was conducted using WSE supplemented with glucose and peptone. In addition, to evaluate whether any of the substances present in wheat straw could affect the growth of the microorganism, SmF was performed using the crude extract obtained from wheat straw and the diluted extract (25% WSE in distilled water). Enzyme production was higher for fermentations with diluted extract (Fig. 2b), which is also related to the lower glucose consumption in the medium with original extract. Maximum activities were reached on day 4 in the fermentation with diluted extract (339.4 ± 11.6 U/L of MnP and 14.8 ± 13.9 U/L of MiP). In the fermentation with original extract (Fig. 2a), the maximum MiP activity was reached on day 4 (10.7 ± 6.1 U/L), and the maximum MnP activity on day 7 (249.0 ± 51.0 U/L).

#### SmF using WSE supplemented with glucose and casein hydrolysate

**Fig. 3:**
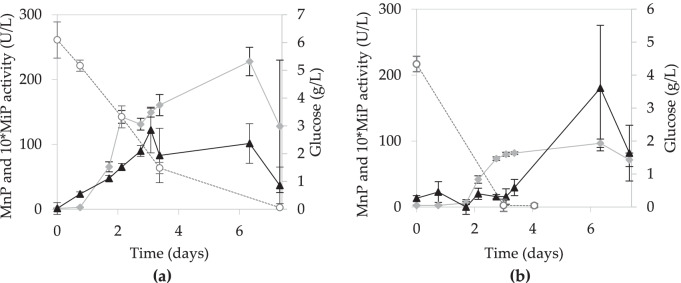
Evolution of MnP activity (  ), MiP activity (▲), and glucose concentration (○) in the SmF with **a** original and **b** diluted WSE supplemented with glucose and casein hydrolysate

It was evaluated whether a change in the type of nitrogen source could affect enzyme production. For this, casein hydrolysate was used as nitrogen source instead of peptone, in addition to the nutrients provided by WSE and glucose. As in the previous fermentations with peptone, both original and diluted WSE were used. Fig. 3 shows that the highest MnP activity was achieved with the medium with the original WSE (227.9 ± 21.9 U/L at day 6), and the activity in the diluted medium was 96.7 ± 6.3 U/L, also at day 6. For MiP activity, the diluted WSE-based medium performed similarly to the medium with the original extract, reaching maximum activities of 18.0 ± 9.5 and 12.2 ± 3.5 U/L after 6 and 3 days, respectively.

#### Scale-up of the SmF to a 30 L reactor

**Fig. 4:**
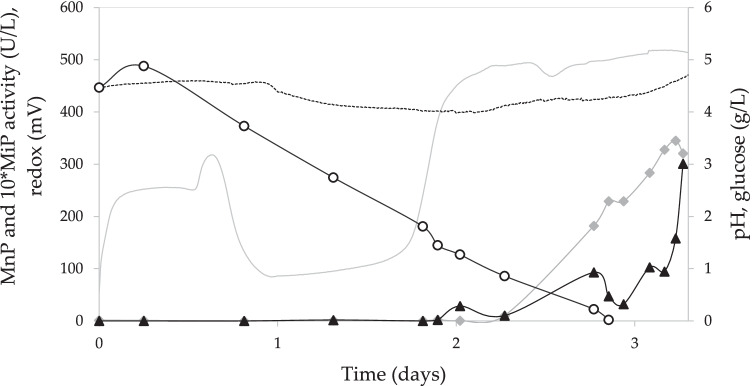
Evolution of pH (- -), redox potential (  ), MnP activity (  ), MiP activity (▲), and glucose concentration (○) during the fermentation in a 30 L fermenter

The SmF with maximum enzyme production was scaled up to a total volume of 30 L, in a stirred tank reactor. During the operation of the reactor, different parameters were measured online (Fig. 4). The pH value decreased to 4.2 after the start of enzyme production, and then increased to 4.9 on day 3, where the maximum MnP activity (345 U/L) was reached. The glucose concentration decreased rapidly in the first days of fermentation, and at the start of enzyme production, it was below 1 g/L. As for the redox potential, it started to increase after enzyme production, and the maximum level was coincident with the maximum enzyme activity.

### Identification of enzyme cocktail proteins

Due to the detection of Mn(II)-dependent and Mn(II)-independent activities, the different proteins present in the concentrated enzyme cocktail were separated using denaturing gel electrophoresis (SDS-PAGE), as a tool to differentiate the enzymes responsible for these activities.

**Fig. 5:**
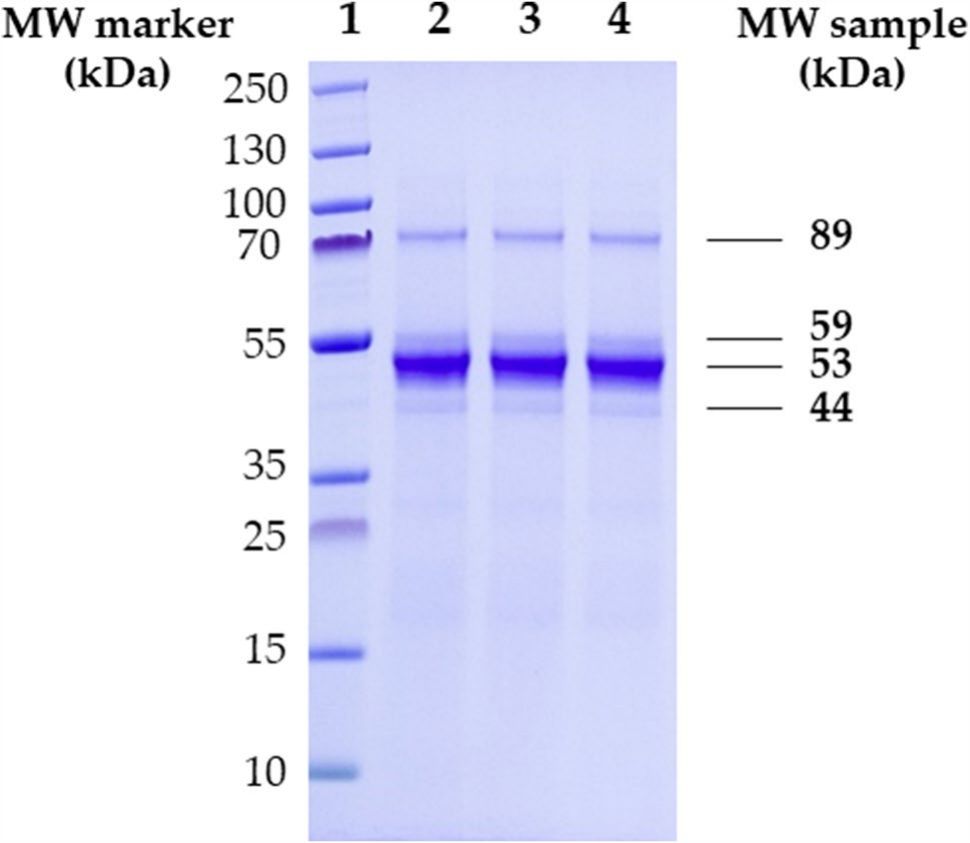
SDS-PAGE of triplicate aliquots from the concentrated enzyme cocktail obtained in the fermentation with *I. lacteus* (lanes 2–4). Molecular weight marker is shown in lane 1

Fig. 5 shows the migration pattern of triplicate cocktail aliquots. The band with the highest intensity corresponds to a molecular weight of 53 kDa, while also other bands with molecular weights of 89, 59, and 44 kDa are visible.

By shotgun proteomic analyses, 18 enzymes were identified in the enzyme cocktail as shown in Supplemental Table S3. Most of them correspond to peroxidases, and among them, interestingly long-chain MnPs, one short-chain MnP, and one DyP. Among the other enzymes present in the enzyme cocktail also proteinases, cellobiohydrolases, and dehydrogenases were detected.

### Removal of micropollutants by enzymatic treatment

Enzymatic removal of CBZ, BPA, E1, E2, and EE2 (Supplemental Table S4) was carried out in batch experiments using the enzyme cocktail produced by *I. lacteus*. The structure and characteristics of these model emerging contaminants are shown in Supplemental Table S4. Control experiments with thermally inactivated enzyme confirmed that H_2_O_2_ by itself, independently of the enzymatic action, could not degrade the ECs (data not shown). Different enzyme activities and H_2_O_2_ concentrations were evaluated, trying to identify the optimal conditions to ensure the highest removal of ECs.

**Fig. 6:**
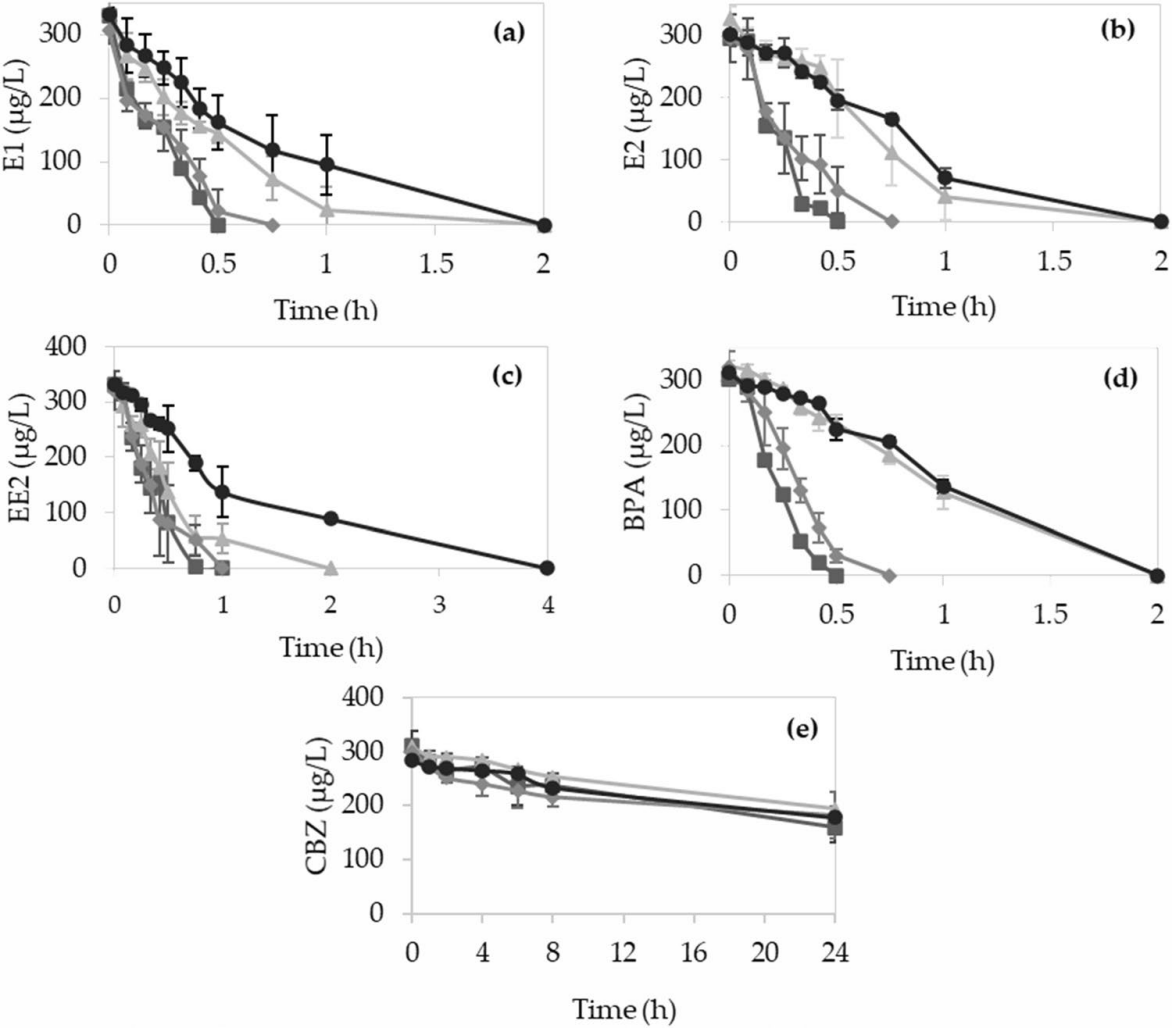
Time course of removal for the target pollutants: **a** E1, **b** E2, **c** EE2, **d** BPA, **e** CBZ in experiments with 200 U/L MnP and 10 µM H_2_O_2_ (  ), 200 U/L MnP and 1 µM H_2_O_2_ (  ), 50 U/L MnP and 10 µM/min H_2_O_2_ (  ), 50 U/L MnP and 1 µM/min H_2_O_2_ (●)

Hormones and bisphenol A were completely biodegraded under all conditions studied. As shown in Fig. 6, the best results were achieved with the highest enzyme activity (200 U/L) and the addition of 10 µM/min H_2_O_2_. Under these conditions, total removal of these compounds was achieved in less than 1 h. For carbamazepine, biodegradation was studied over 24 h, with maximum degradation between 12.5 (lowest H_2_O_2_ supply) and 28.8% (highest H_2_O_2_ supply and highest enzyme activity).

Table 3 shows the apparent first-order kinetic constants for each EC studied. These results show that higher concentrations of H_2_O_2_ improved the biodegradation for all the compounds studied. In fact, the highest apparent kinetic constants (> 2 h^−1^) corresponded to experiments with 10 µM/min H_2_O_2_. The enzymatic dose also had an effect on the biodegradation kinetics. Comparing experiments with the same H_2_O_2_ addition rate, decreasing the initial activity from 200 to 50 U/L resulted in decreased apparent kinetic constant values (from 8 to 55% lower values). It is also remarkable that the decrease in enzyme activity (MnP and MiP) after 24 h was always less than 15%.

**Table 3:** Conditions and results of ECs degradation with different enzyme doses and H_2_O_2_ concentration

Compound	Initial activity (U/L)		24 h activity (U/L)		H_2_O_2_ (µM/min)	Time (h)	Degradation (%)	*k*^a^ (h^−1^)	*r*^2^
	MnP	MiP	MnP	MiP					
E1	193 ± 1	123 ± 7	168 ± 3	121 ± 12	10	0.5	100	3.5	0.95
	202. ± 8	143 ± 11	188 ± 4	134 ± 8	1	2	100	1.9	0.98
	48 ± 3	30 ± 2	48 ± 2	26 ± 1	10	0.75	100	2.5	0.93
	45 ± 2	29 ± 7	43 ± 7	27 ± 3	1	2	100	1.1	0.97
E2	193 ± 1	123 ± 7	168 ± 3	121 ± 12	10	0.5	100	6.4	0.82
	202 ± 8	143 ± 11	188 ± 4	134 ± 8	1	2	100	0.70	0.90
	48 ± 3	30 ± 2	48 ± 2	26 ± 1	10	0.75	100	3.4	0.97
	45 ± 2	29 ± 7	43 ± 7	27 ± 3	1	2	100	0.59	0.91
EE2	193 ± 1	123 ± 7	168 ± 3	121 ± 12	10	1	100	2.6	0.97
	202 ± 8	143 ± 11	188 ± 4	134 ± 8	1	2	100	1.3	0.92
	48 ± 3	30 ± 2	48 ± 2	26 ± 1	10	1	100	2.4	0.96
	45 ± 2	30 ± 7	43 ± 7	27 ± 3	1	4	100	0.59	0.92
BPA	193 ± 1	123 ± 7	168 ± 3	121 ± 12	10	0.5	100	5.2	0.91
	202 ± 8	143 ± 11	188 ± 4	134 ± 8	1	2	100	0.63	0.90
	48 ± 3	30 ± 2.0	48 ± 2	26 ± 1	10	0.75	100	2.60	0.93
	45 ± 2	29 ± 7	43 ± 7	27 ± 3	1	2	100	0.37	0.93
CBZ	193 ± 1	123 ± 7	168 ± 3	121 ± 12	10	24	28.8	0.03	0.96
	202 ± 8	143 ± 11	188 ± 4	134 ± 8	1	24	12.5	0.02	0.99
	48 ± 3	30 ± 2.0	48 ± 2	26 ± 1	10	24	15.1	0.02	0.83
	45 ± 2	29 ± 7	43 ± 7	27 ± 3	1	24	12.5	0.02	0.98

^a^Apparent kinetic constant according to first-order kinetics

## Discussion

As demonstrated in the experimental results corresponding to the characterization of the enzyme cocktail, MnP activity was obtained in all the fermentations performed, thus confirming that wheat straw is a suitable substrate for producing MnP enzymes. In addition, MiP activity was also determined, with lower values than those obtained for MnP, in agreement with other reports on *I. lacteus* (Salvachúa et al. 2013b). Specifically, the maximum levels of MnP activity are approximately 10 times higher than the maximum levels of MiP activities. The fact that wheat straw contains significant levels of manganese (50 mg/L) (Hofrichter et al. 1999) leads to more suitable conditions that favor MnP production. Laccase activity was also measured in all fermentations with wheat straw-based media, but no laccase activity was detected in any of the trials.

In relation to the results obtained in the SSFs, it is important to note that the best enzyme production occurred in the non-supplemented medium. Several studies have demonstrated that low N and C concentrations in SSF can stimulate secondary metabolism in WRF, which is responsible of ligninolytic enzyme production (Elisashvili et al. 2008; Iqbal et al. 2011). Concretely, Elisashvili et al. (2008) have established that nitrogen supplementation in SSF with wheat straw had a negative impact on enzyme production by *Pleurotus ostreatus*. It is likely that the fungus first consumes easily metabolizable nutrients, and then initiates the secretion of ligninolytic enzymes as a tool or strategy to access the C source present in wheat straw (Elisashvili et al. 2008). This fungus presents a high capacity to degrade acid-insoluble lignin (Zuo et al. 2018), which is the most abundant type of lignin present in the wheat straw used in this study (see Supplemental Table S1). This ability to degrade lignocellulosic material present in the deeper layers of wheat straw is directly related to a higher production of ligninolytic enzymes.

The pretreatment of wheat straw used in SSF also influences enzyme production. In this study, the maximum production in the NS-SSF was reached on day 9. However, SSF studies using wheat straw that was not subjected to any pretreatment showed different fermentation periods to reach maximum MnP activity, varying from 14 to 23 days (Dias et al. 2010; Salvachúa et al. 2013b). Nevertheless, SSF studies using other agro-industrial wastes, such as olive biomass, have shown longer fermentation times (30 days) to reach maximum enzyme production (Martínez-Patiño et al. 2018). Despite the potential of enzyme production in SSF, the time period required to obtain maximum production is too long, which was one of the reasons why the production of ligninolytic enzymes in SmF was addressed. First, SmFs were performed on conventional media to have a reference of the enzyme production of *I. lacteus* and to evaluate the impact of the use of WSE in SmF. For SmFs with WSE, other sources of nitrogen and carbon were added to ensure a nutrient-balanced medium for an optimal fungal growth. Thus, in addition to glucose supplementation to increase the concentration of fermentable sugars, two alternatives for the nitrogen source were considered: peptone or hydrolyzed casein (Elisashvili et al. 2008). The choice of organic nitrogen sources over inorganic ones was based on previous studies that reported that organic compounds perform better than inorganic compounds in fungal SmF, due to their ability to stimulate fungal growth (Juwon and Emmanuel 2012). As can be seen in Fig. 2, the use of peptone stimulated enzyme production (Lú-Chau et al. 2018; Pinheiro et al. 2020).

The fermentation using 100% WSE supplemented with glucose and peptone (Fig. 2a) showed lower glucose consumption than the fermentation with 25% WSE. The presence of potential inhibiting compounds in the wheat straw extract may be affecting the fungal growth and hence glucose consumption. Interestingly, the production of enzymes when using 100% WSE was faster (activity started on day 2 compared to day 4 in diluted extract), which could be due to dissolved lignin derived compounds acting as inducers of ligninolytic enzymes production. This effect of aromatic compounds acting as inducers has been previously described, and it was suggested that ligninolytic enzyme synthesis may function as a defense mechanism against chemical stress (Elisashvili et al. 2010). Although the production of enzymes was faster in the original extract and maintained for a longer period, the maximum production was achieved in the diluted extract after 4 days of fermentation.

In the case of SmF supplemented with casein hydrolysate, the trend was similar, showing earlier production of enzymes in the medium with original extract, but in this case, it also showed the highest enzyme production.

It should be noted that all SmFs with WSE-based medium showed similar or higher enzyme production than SmFs in conventional medium, which makes WSE a good alternative for the production of ligninolytic enzymes, with the advantage that it is possible to valorize an agricultural waste in the context of a circular economy approach.

MnP production under the best conditions in SmF was successfully scaled up to a 30 L reactor, obtaining a MnP activity of 345 U/L on the third day of fermentation. In addition, the analysis of the redox potential and pH profile allows to determine an indicator of increased enzyme production. As shown in Fig. 4, before the onset of enzyme production, the pH value decreased to around 4.4 and after the peak of MnP production, the pH increased again. On the other hand, the redox potential underwent an increase as more enzyme was produced, reaching its maximum value (500 mV) in parallel to the peak of enzyme production (Lú-Chau et al. 2018). Another remarkable fact is that enzyme production started once the glucose concentration was below 0.8 g/L, similar to the trend observed in Erlenmeyer flask experiments. This is mainly because low glucose levels induce a phase shift from primary to secondary metabolism, a phase in which the fungus begins to synthesize ligninolytic enzymes (Iqbal et al. 2011).

The production of enzyme cocktails with MnP and MiP activities has been documented for different WRF species (Chen et al. 2015; Duan et al. 2018). However, very few articles analyze whether this activity is due to a single enzyme or to a set of enzymes. The expression of a short manganese peroxidase with both MnP and MiP activity has already been described for *I. lacteus* (Li et al. 2019). Therefore, it is essential to determine its presence in the obtained enzyme cocktail, as it could offer some advantages due to its shorter C-terminal length that can increase the catalytic properties by a better interaction with the substrate molecule (Li et al. 2019).

Considering the SDS-PAGE electrophoresis results, it is clear that the proteins present in highest abundance correspond to a molecular weight (MW) of 53 kDa. However, in shotgun proteomic analyses, the most abundant proteins were MnPs with MWs ranging from 37.25 to 40.25 kDa. This is in agreement to Shin et al. (2005), who reported a purified MnP from *I. lacteus* with a MW of 38.3 according to their MALDI-TOF analyses, while in SDS-PAGE, its band was located in 53 kDa. Also, in Wang et al. (2002), an isolated MnP from *Bjerkandera adusta* showed an apparent MW in SDS-PAGE of 43 kDa in SDS-PAGE and 36.6 kDa by MALDI-TOF. These differences can be explained by the fact that MnPs are glycosilated proteins, and high carbohydrate contents can affect migration in SDS-PAGE. Another band observed in the gel at 44 kDa might correspond to a short MnP. Its MW is in agreement with other values reported in the literature for short MnPs of *I. lacteus* in SDS-PAGE (43–45 kDa) (Chen et al. 2015; Duan et al. 2018). In this case, as the intensity of the band is much lower than that of the main MnP (4 times lower), we assume that its involvement in the degradation of the ECs is lower than that of the main MnP. By mass spectrometry, one enzyme identified with 5 unique peptides (Supplemental Table S5) corresponds with a short MnP of *I. lacteus* previously characterized by Chen et al. (2015) (GenBank: AGO86670.2).

On the other hand, the presence of a band at the MW of 59 kDa could correspond to a dye-decolorizing peroxidase (DyP). Salvachúa et al. (2013a) have already described a DyP produced with *I. lacteus* on wheat straw with a molecular weight of 57 kDa in SDS-PAGE. The presence of this enzyme together with short MnP would justify the MiP activity measured in the enzyme cocktail. Accordingly, proteomic analysis detected a DyP with 17 unique peptides (Supplemental Table S6). Other enzymes were detected in the enzyme cocktail, including proteinases, cellobiohydrolases, and dehydrogenases. A weak band is observed in SDS-PAGE at a corresponding MW of 89 kDa which could not be explained by any of the proteins identified by shotgun proteomics. One possibility is that this band is composed by the choline dehydrogenases detected in shotgun with MW of 65.87 kDa. But it cannot be discarded that this might be the result of the artificial dimerization of an enzyme during the sample processing for SDS-PAGE, which is rare but have already been described, especially for highly hydrophobic proteins (Rath et al. 2009).

Regarding the abovementioned, the proteomic analysis confirmed the presence of different peroxidases in the enzyme cocktail and is a more precise tool for the identification of enzymes and their corresponding MW in mixed cocktails.

The enzymatic cocktail produced is considered a suitable biocatalyst for the degradation of the ECs studied. Bisphenol A and hormones were degraded in less than 1 h for the highest dose of H_2_O_2_. This shows that H_2_O_2_ concentration is a key factor for the enzyme activity, since when a lower dose was used, twice as much time was needed to obtain a complete degradation of these compounds. This correlation between the H_2_O_2_ dose and the improvement of the degradation kinetics of aromatic pollutants with peroxidases has been reported in different studies (Eibes et al. 2011; Alneyadi et al. 2017). In none of the controls carried out with the inactivated enzyme, degradation of the compounds was observed, which rules out the possibility that H_2_O_2_ alone could transform the ECs.

However, H_2_O_2_ concentration should be a parameter to control, since an excess of hydrogen peroxide can lead to irreversible damage of the heme group, a process known as suicidal inactivation (Valderrama et al. 2002). Apparently, the feed rates used in the present work did not lead to an excess of H_2_O_2_, since losses of activity lower than 13% were measured after 24 h of reaction. Therefore, the enzyme cocktail could be reused in consecutive cycles or even in continuous process, using an ultrafiltration membrane to retain the enzyme in the reactor (Lloret et al. 2013).

The addition of the enzyme had an effect on the degradation kinetics of the compounds, particularly evident for hormones and BPA. Although the antiepileptic CBZ was more recalcitrant than the other compounds, the highest apparent kinetic constant was obtained with the highest addition of peroxide and enzyme. It might be interesting to evaluate degradation at even higher concentrations, although Eibes et al. (2011) reported that there was no significant removal of CBZ, even by increasing the initial VP activity to 1000 U/L.

The degradation times are similar for BPA, E1, and E2, if the results are compared under the same conditions. However, in the case of EE2, despite having a similar structure to the other two hormones, the degradation in some experiments was slower. This could be due to the presence of an ethinyl group in its structure (shown in Supplemental Table S4), which can make EE2 more resistant to degradation (Nejedly and Klimes 2017).

In the case of carbamazepine, a high reaction time is necessary to achieve a partial degradation of 30%. To our knowledge, this is the first work evaluating carbamazepine degradation with crude MnP. Studies performed with versatile peroxidase and lignin peroxidase either did not achieve carbamazepine degradation or it was less than 10% (Zhang and Geißen 2010; Eibes et al. 2011). The higher degradation achieved in the present study may be due to the presence of DyP and short MnP, which can act on the same substrates as the main MnP. In this regard, it has been reported that some DyP of WRF may also have catalytic efficiency in the oxidation of Mn^2+^ at pH 4.5 (Fernández-Fueyo et al. 2015), which could have a slight impact on the degradation of carbamazepine. Furthermore, although the catalytic efficiency towards Mn^2+^ of short MnP may be lower than that of other MnPs, it is able to oxidize other substrates (such as ABTS and different phenols) (Fernández-Fueyo et al. 2015).

In order to achieve a complete carbamazepine elimination, the enzymatic treatment must be combined with other techniques, such as photocatalytic treatment. In this regard, Calza et al. (2016) found a synergistic effect of photocatalysis and soybean peroxidase (SBP), obtaining a complete degradation of carbamazepine within 60 min, whereas SBP alone was not effective. However, advanced oxidation processes can result in the transformation of carbamazepine into compounds with higher toxicity potential (Mohapatra et al. 2014), so any degradation process has to be supported by lower environmental impact and toxicity.

Summarizing, wheat straw is a great alternative substrate to support the growth of *I. lacteus* and to induce the production of ligninolytic enzymes in both solid-state and submerged fermentations. It is also demonstrated that the supply of different types of supplementary nitrogen sources has a significant effect on the enzymatic activities obtained in the submerged state. Most studies evaluating lignocellulosic residues for fungal fermentation have achieved the production of enzyme cocktails with Mn(II)-dependent activity, but there are a few studies reporting the production of an enzyme cocktail with both Mn(II)-dependent and Mn(II)-independent activities. The enzymatic crude was suitable for complete removal of bisphenol A, estrone, 17b-estradiol, and 17a-ethinylestradiol and partial removal of carbamazepine. Based on the results obtained, it can be concluded that the use of the set of enzymes produced by *I. lacteus* in wheat straw extract is a good alternative to remove emerging pollutants with a similar structure to the studied compounds from wastewater.

## Supplementary Information

Below is the link to the electronic supplementary material.

Supplementary file1 (PDF 456 KB)

## Data Availability

The data used to support the findings of this study are available from the corresponding author upon reasonable request.
