# Supplementary material for: Bundling the removal of emerging contaminants with the production of ligninolytic enzymes from residual streams
Source: Appl Microbiol Biotechnol. 2022 Jan 25;106(3):1299–311. doi: 10.1007/s00253-022-11776-7 (PMC8816780; doi:10.1007/s00253-022-11776-7)
Supplement: Supplementary file 1 — Supplementary file1 (PDF 456 KB) [file 253_2022_11776_MOESM1_ESM.pdf]

Applied Microbiology and Biotechnology

Supplementary information

Bundling the removal of emerging contaminants with the production of ligninolytic enzymes from residual streams

Sandra González-Rodríguez*, Thelmo A. Lu-Chau, Alba Trueba-Santiso, Gemma Eibes and María Teresa Moreira

CRETUS, Department of Chemical Engineering, Universidade de Santiago de Compostela, 15782 Santiago de Compostela, Galicia, Spain
* Correspondence: s.gonzalez.rodriguez@usc.es; Tel.: +34881816014

Supplemental Table S1. Wheat straw composition.

**Total lignin (%)**	49.94±0.52
**Acid insoluble lignin (%)**	44.90±1.24
**Acid-soluble lignin (%)**	5.59±0.36
**Total reducing sugars (%)**	9.4±0.2
**Ash (%)**	0.547±0.365

Supplemental Table S2. Characteristics of concentrated wheat straw extract.

**pH**	6.66
**Total nitrogen (mg/L)**	69.4±0.3
**Total organic carbon (mg/L)**	924.2±4.1
**Glucose (mg/L)**	92±16
**Total reducing sugars (mg/L)**	224±8

Supplemental Table S3. List of proteins identified in the shotgun proteomics analysis of the enzyme cocktail from *I. lacteus*. Proteins identified with ⩽2 unique peptides were not taken into account. Proteins are ranked by their Spec number, calculated with Peaks software and which can be correlated with their abundance in the sample.

NCBI Accession	Description	Area	Nº Peptides	Nº Unique Peptides	Spec Number	Avg. Mass (kDa)	-10lgP	Coverage (%)
**AQT03613.1**	manganese peroxidase 2	4.45E+04	28	5	83	37.8	298.08	45
**AGO86670.2**	short manganese peroxidase	4.45E+04	28	5	83	37.83	298.08	45
**AZJ17947.1**	manganese peroxidase	1.37E+05	22	20	54	37.94	296.31	50
**AZJ17945.1**	manganese peroxidase	1.37E+05	22	20	54	38.24	296.31	49
**ANA52681.1**	dye-decolorizing peroxidase partial	1.01E+05	17	17	49	48.42	253.26	29
**AZJ17942.1**	manganese peroxidase	3.26E+04	5	3	28	30.32	138.89	21
**AZJ17939.1**	manganese peroxidase	1.99E+04	5	4	19	37.25	158.4	15
**sp|P17576.1|CARP_IRPLA**	RecName: Full=Polyporopepsin	3.60E+04	7	7	18	35.05	176.35	14
**prf||1512141A**	Asp protease	3.60E+04	7	7	18	35.05	176.35	14
**BAA00467.1**	aspartic proteinase precursor partial	3.60E+04	7	7	18	35.05	176.35	14
**pdb|1WKR|A**	Chain A Polyporopepsin	3.60E+04	7	7	18	35.04	176.35	14
**AQT03612.1**	manganese peroxidase 1	1.26E+04	6	6	13	37.92	153.92	16
**AZJ17938.1**	manganese peroxidase	1.26E+04	6	6	13	40.25	153.92	15
**AZJ17940.1**	manganese peroxidase	2.11E+02	5	3	12	37.96	125.02	14
**BAD16575.1**	cellobiohydrolase	5.86E+02	6	6	7	54.84	159.3	11
**ALJ82906.1**	mitochondrial choline dehydrogenase	9.06E+01	4	4	5	65.87	134.37	9
**ALJ82895.1**	choline dehydrogenase	8.92E+02	3	3	4	65.87	112.04	6
**BAG48183.1**	cellobiohydrolase II	4.55E+02	2	2	2	47.28	91.53	5

Supplemental Table S4: Characteristics of studied ECs.

Compound	Molecular weight (Da)	Water solubility (mg/L)	Structure
Estrone (E1)	270.4	30	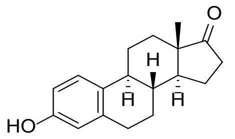
17β-estradiol (E2)	272.4	3.6	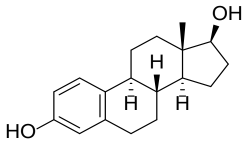
17α-ethinylestradiol (EE2)	296.4	11.3	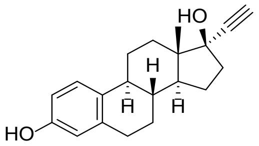
Bisphenol A (BPA)	228.3	12	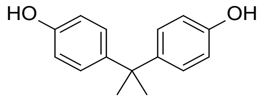
Carbamazepine (CBZ)	236.3	17.7	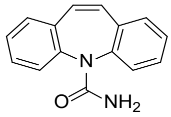

Supplemental Table S5. Protein coverageand list of supporting peptides for the identified short manganese peroxidase (MnP, NCBI accession: AGO86670.2).

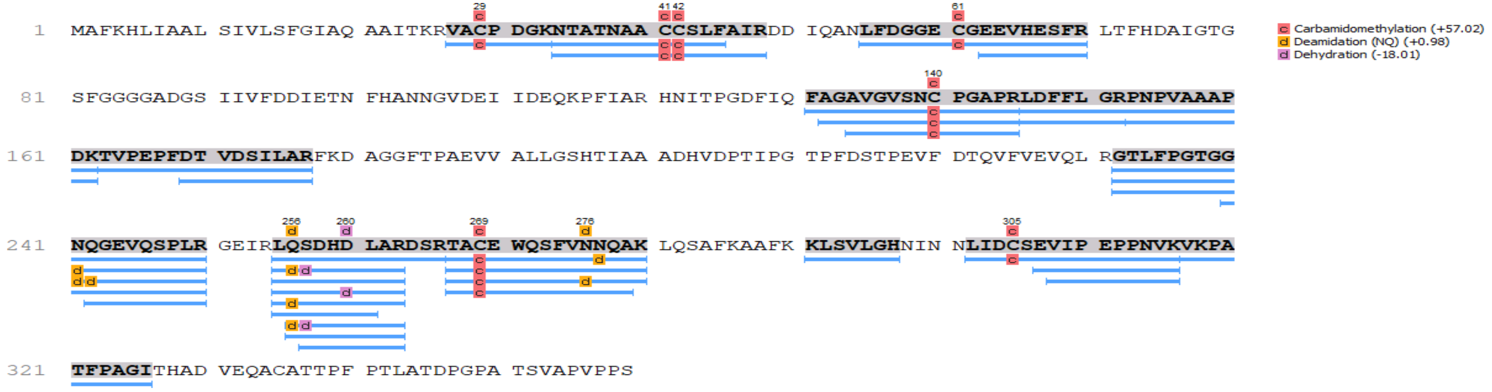

Supporting Peptides:

Peptide	Uniq	-10lgP	Mass	Length	ppm	m/z	z	RT	1/k0 Range	Fraction	Precursor Id	Source File	Area CE_MS955	#Feature	#Feature CE_MS955	Start	End	PTM	AScore	Found By
R.TAC(+57.02)EWQSFVNNQAK.L	N	100.01	1681.7518	14	1.4	841.8844	2	13.94	1.0581-1.0805	6	9152	CE_bis_MS955_Slot1-5_1_664.d	1.3832E4	2	2	267	280	Carbamidomethylation	C3:Carbamidomethylation:1000.00	PEAKS DB
R.GTLFPGTGGNQGEVQSPLR.G	Y	84.96	1913.9595	19	3.4	957.9902	2	14.55	1.1180-1.1402	6	9758	CE_bis_MS955_Slot1-5_1_664.d	1.1662E4	1	1	232	250			PEAKS DB
Q.FAGAVGVSNC(+57.02)PGAPR.L	N	83.22	1458.7037	15	0.3	730.3594	2	10.12	0.9872-1.0097	6	5177	CE_bis_MS955_Slot1-5_1_664.d	1.5834E3	1	1	131	145	Carbamidomethylation	C10:Carbamidomethylation:1000.00	PEAKS DB
R.TAC(+57.02)EWQSFVN(+.98)NQAK.L	N	78.24	1682.7358	14	-8.9	842.3677	2	14.21	1.0635-1.0858	6	9438	CE_bis_MS955_Slot1-5_1_664.d	0	0	0	267	280	Carbamidomethylation; Deamidation (NQ)	C3:Carbamidomethylation:1000.00;N10:Deamidation (NQ):12.28	PEAKS DB
N.LIDC(+57.02)SEVIPEPPNVK.V	N	77.16	1708.8705	15	1.1	855.4435	2	14.58	1.0697-1.0921	6	9854	CE_bis_MS955_Slot1-5_1_664.d	4.5797E3	1	1	302	316	Carbamidomethylation	C4:Carbamidomethylation:1000.00	PEAKS DB
R.TAC(+57.02)EWQSFVNN(+.98)QAK.L	N	70.07	1682.7358	14	6.2	842.3804	2	13.85	1.0492-1.0715	6	8938	CE_bis_MS955_Slot1-5_1_664.d	1.0151E3	1	1	267	280	Carbamidomethylation; Deamidation (NQ)	C3:Carbamidomethylation:1000.00;N11:Deamidation (NQ):0.00	PEAKS DB
K.NTATNAAC(+57.02)C(+57.02)SLFAIR.D	N	67.27	1668.7712	15	2.8	835.3952	2	14.74	1.0492-1.0715	6	10135	CE_bis_MS955_Slot1-5_1_664.d	4.1538E2	1	1	34	48	Carbamidomethylation	C8:Carbamidomethylation:1000.00;C9:Carbamidomethylation:1000.00	PEAKS DB
R.PNPVAAAPDK.T	Y	66.34	978.5134	10	2.8	490.2654	2	6.90	0.7927-0.8154	6	1994	CE_bis_MS955_Slot1-5_1_664.d	5.3618E3	1	1	153	162			PEAKS DB
K.TVPEPFDTVDSILAR.F	N	66.02	1658.8516	15	-0.2	830.4329	2	16.55	1.0876-1.1099	6	12111	CE_bis_MS955_Slot1-5_1_664.d	1.3849E3	1	1	163	177			PEAKS DB
R.LQSDHDLAR.D	N	66.02	1053.5203	9	-2.3	527.7662	2	5.78	0.8690-0.8916	6	1049	CE_bis_MS955_Slot1-5_1_664.d	1.4065E5	3	3	255	263			PEAKS DB
R.LDFFLGRPNPVAAAPDK.T	Y	65.94	1826.9678	17	2.9	609.9983	3	15.19	0.8517-0.8744	6	10681	CE_bis_MS955_Slot1-5_1_664.d	2.2288E2	1	1	146	162			PEAKS DB
R.GTLFPGTGGN(+.98)QGEVQSPLR.G	Y	65.80	1914.9435	19	5.1	958.4839	2	14.69	1.1224-1.1447	6	10291	CE_bis_MS955_Slot1-5_1_664.d	2.7007E4	1	1	232	250	Deamidation (NQ)	N10:Deamidation (NQ):23.70	PEAKS DB

Total 36 peptides

Supplemental Table S6. Protein coverageand list of supporting peptides for the identified dye-decolorizing peroxidase (DyP, NCBI accession: ANA52681.1).

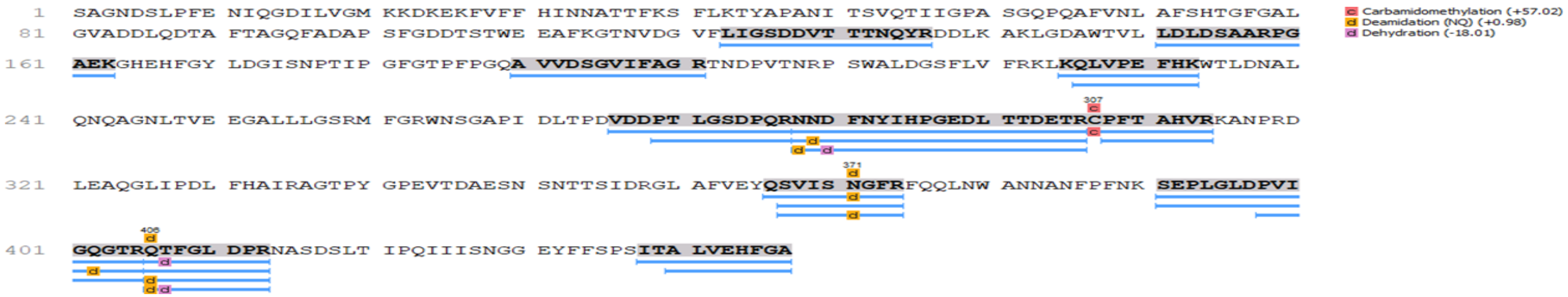

Supporting Peptides:

Peptide	Uniq	-10lgP	Mass	Length	ppm	m/z	z	RT	1/k0 Range	Fraction	Precursor Id	Source File	Area CE_MS955	#Feature	#Feature CE_MS955	Start	End	PTM	AScore	Found By
R.NNDFNYIHPGEDLTTDETR.C	Y	103.44	2249.9824	19	3.8	751.0043	3	13.63	0.8871-0.9097	6	8749	CE_bis_MS955_Slot1-5_1_664.d	4.2392E3	2	2	288	306			PEAKS DB
K.SEPLGLDPVIGQGTR.Q	Y	77.68	1537.8099	15	-0.2	769.9121	2	14.74	0.9944-1.0169	6	10062	CE_bis_MS955_Slot1-5_1_664.d	1.3605E4	1	1	391	405			PEAKS DB
R.NN(+.98)DFNYIHPGEDLTTDETR.C	Y	74.39	2250.9666	19	9.0	751.3362	3	14.21	0.8853-0.9079	6	9418	CE_bis_MS955_Slot1-5_1_664.d	0	0	0	288	306	Deamidation (NQ)	N2:Deamidation (NQ):0.00	PEAKS DB
R.C(+57.02)PFTAHVR.K	Y	66.87	986.4756	8	-0.8	494.2447	2	7.26	0.8272-0.8499	6	2213	CE_bis_MS955_Slot1-5_1_664.d	7.874E3	2	2	307	314	Carbamidomethylation	C1:Carbamidomethylation:1000.00	PEAKS DB
K.QLVPEFHK.W	Y	66.20	996.5392	8	1.5	499.2776	2	10.43	0.8327-0.8554	6	5430	CE_bis_MS955_Slot1-5_1_664.d	2.0342E4	2	2	226	233			PEAKS DB
R.QTFGLDPR.N	Y	58.59	932.4716	8	0.7	467.2434	2	11.01	0.8136-0.8363	6	5944	CE_bis_MS955_Slot1-5_1_664.d	5.3749E4	1	1	406	413			PEAKS DB
Q.AVVDSGVIFAGR.T	Y	56.81	1189.6455	12	-3.3	595.8281	2	14.29	0.8979-0.9205	6	9536	CE_bis_MS955_Slot1-5_1_664.d	0	0	0	190	201			PEAKS DB
T.ALVEHFGA	Y	56.67	842.4286	8	-0.7	422.2213	2	11.21	0.7735-0.7963	6	6218	CE_bis_MS955_Slot1-5_1_664.d	7.1547E2	1	1	440	447			PEAKS DB
D.VDDPTLGSDPQR.N	Y	55.60	1298.6102	12	-2.7	650.3107	2	7.96	0.9142-0.9368	6	2871	CE_bis_MS955_Slot1-5_1_664.d	0	0	0	276	287			PEAKS DB
L.KQLVPEFHK.W	Y	55.19	1124.6342	9	7.5	375.8882	3	8.28	0.7178-0.7406	6	3191	CE_bis_MS955_Slot1-5_1_664.d	0	0	0	225	233			PEAKS DB
R.Q(+.98)TFGLDPR.N	Y	52.54	933.4556	8	2.3	467.7361	2	11.58	0.8118-0.8345	6	6618	CE_bis_MS955_Slot1-5_1_664.d	9.3235E1	1	1	406	413	Deamidation (NQ)	Q1:Deamidation (NQ):1000.00	PEAKS DB
Q.SVISN(+.98)GFR.F	Y	51.96	879.4450	8	-0.2	440.7297	2	9.96	0.7726-0.7954	6	5082	CE_bis_MS955_Slot1-5_1_664.d	0	0	0	367	374	Deamidation (NQ)	N5:Deamidation (NQ):1000.00	PEAKS DB
R.QT(-18.01)FGLDPR.N	Y	51.15	914.4610	8	-0.5	458.2375	2	11.01	0.8054-0.8281	6	6069	CE_bis_MS955_Slot1-5_1_664.d	0	0	0	406	413	Dehydration	T2:Dehydration:42.68	PEAKS DB
D.PTLGSDPQR.N	Y	50.63	969.4879	9	9.6	485.7559	2	6.46	0.8008-0.8236	6	1777	CE_bis_MS955_Slot1-5_1_664.d	0	0	0	279	287			PEAKS DB
Y.QSVISN(+.98)GFR.F	Y	50.51	1007.5036	9	0.2	504.7592	2	10.21	0.8218-0.8445	6	5266	CE_bis_MS955_Slot1-5_1_664.d	1.2676E2	1	1	366	374	Deamidation (NQ)	N6:Deamidation (NQ):76.17	PEAKS DB
F.LIGSDDVTTTNQYR.D	Y	50.27	1581.7634	14	-0.9	791.8882	2	10.28	1.0178-1.0402	6	5353	CE_bis_MS955_Slot1-5_1_664.d	0	0	0	123	136			PEAKS DB
C.PFTAHVR.K	Y	49.53	826.4449	7	6.3	276.4907	3	6.43	0.6618-0.6848	6	1769	CE_bis_MS955_Slot1-5_1_664.d	0	0	0	308	314			PEAKS DB

Total 24 peptides
